# Citrate treatment reduces endothelial death and inflammation under hyperglycaemic conditions

**DOI:** 10.1177/1479164111424297

**Published:** 2012-01

**Authors:** Anna Bryland, Anders Wieslander, Ola Carlsson, Thomas Hellmark, Gabriela Godaly

**Affiliations:** 1 Department of Nephrology, Lund University, Lund, Sweden; 2 Gambro Lundia AB, Lund, Sweden; 3 Department of Microbiology, Immunology and Glycobiology, Lund University, Lund, Sweden

**Keywords:** Apoptosis, diabetes, endothelium, hyperglycaemia, inflammation

## Abstract

Hyperglycaemia and glucose degradation products (GDPs) are closely associated with oxidative stress and inflammation in diabetic patients, a condition that leads to endothelial dysfunction and cardiovascular problems. We evaluated the effect of citrate and gluconate on glucose- and GDP-induced endothelial inflammation by measuring changes in viability, inflammation and function in primary human umbilical vein endothelial cells (HUVECs). The extent of apoptosis/necrosis was measured by flow cytometry and visualised with confocal microscopy by staining with annexin V or propidium iodide, respectively. Protein kinase C-βII (PKC-βII) activation was evaluated with Western blotting. Incubation with glucose (30 mM) and GDP (50 µM) significantly increased PKC-βII expression, endothelial cell death and inflammation. The addition of citrate decreased hyperglycaemia-induced apoptosis (*p* = 0.021), necrosis (*p* = 0.04) and reduced PKC-βII expression (*p* = 0.021) down to background levels. Citrate improved endothelial function by reducing the inflammatory markers (*p* = 0.01) and by decreasing neutrophil diapedesis (*p* = 0.012). These results suggest that citrate may have therapeutic potential by reducing hyperglycaemia-induced endothelial inflammation and abolishing endothelial dysfunction.

## Introduction

Diabetic patients have a high risk of hyperglycaemia, a condition that is considered to be a major cause of cardiovascular disease (CVD).^1^ Diabetes is characterised by the development of specific micro- and macrovascular complications and by high incidence of accelerated atherosclerosis.^2^ Hyperglycaemia-caused endothelial dysfunction plays a key role in the pathogenesis of diabetes complications and numerous studies have linked the activation of protein kinase C (PKC) to the diabetic state.^3^ PKC activation regulates vascular permeability and is involved in endothelial cell activation, abnormal angiogenesis, synthesis of the extracellular matrix, excessive apoptosis, leukocyte adhesion and cytokine production.^4^

In addition to glucose itself, increased vascular atherosclerosis is further caused by the degradation products (GDPs) of glucose.^5^ Reactive GDPs, such as 3,4-dideoxyglucosone-3-ene (3,4-DGE), react with proteins to form advanced glycation end products (AGEs) in the circulation.^6–8^

Severely hyperglycaemic patients typically suffer from complications such as infections and decreased wound healing.^9^ Postoperative hyperglycaemia is also a recognised risk factor for postoperative surgical site infections that leads to increased rehospitalisation, prolonged length of hospital stay and increased health-care costs.^10^ Highly reactive GDPs, such as 3,4-DGE, react instantly with different molecules, while others, such as 3-deoxyglucosone (3-DG), remain in the circulation.^11^ The amount of 3-DG in the serum of a healthy human is low, but increases two-fold with disease such as diabetes and threefold in uraemia.^12^ Furthermore, 3-DG is converted to the more reactive 3,4-DGE through equilibrium.^13^ 3,4-DGE was recently demonstrated to decrease neutrophil viability, impair immune responses and decrease bacterial killing.^8^ Hyperglycaemia and GDPs are further known to induce caspase-mediated apoptosis and oxidative stress in endothelial cells and neutrophils.^8,14,15^ AGE activation of endothelial cells stimulates a host of proinflammatory events, such as the secretion of tumour necrosis factor alpha (TNFα) and interleukin-6 (IL-6), but also the induction of chemotactic cytokines such as interleukin-8 [IL-8; chemokine (C-X-C motif) ligand 8 (CXCL8)] and chemokine (C-C motif) ligand 2 (CCL2; MCP-1), leading to increased endothelial inflammation and leukocyte recruitment.^16,17^ Hyperglycaemia-induced reactive oxygen species (ROS) production leads also to upregulation of the intercellular adhesion molecule-1 (ICAM-1) and vascular cellular adhesion molecule-1 (VCAM-1).^18^ The sum of these events leads to adhesion and migration of leukocytes across endothelial cells, which play an important role in the development of atherosclerosis.^19^

Citrate is an intermediate in the citric acid cycle and is widely used in the food and drug industry because of its buffer, anticoagulant and antioxidant capacities. As a chelator, citrate is able to bind calcium and metals that catalyse the production of ROS. Different chelators have been evaluated against the development of diabetic complications in numerous *in vivo* studies, but the results are not uniformly positive.^20–22^ Citrate addition during dialysis was shown to improve clinical parameters and to decrease inflammation,^23–25^ but there are no studies published on citrate treatment during hyperglycaemic conditions. Another antioxidant with chelating properties is gluconate, which was recently shown to improve endothelial function.^26^ Although the clinical usage of citrate is gaining popularity, in-depth knowledge about its anti-inflammatory mechanisms are unknown. The aim of this study was to investigate the anti-inflammatory capacity of citrate and the combination of citrate and gluconate on hyperglycaemia- or 3,4-DGE-damaged endothelial cells.

## Methods

### Cell culture

Primary human umbilical vein endothelial cells (HUVECs) (Clonetics; Lonza Cologne GmbH, Cologne, Germany) were obtained and cultured in endothelial cell growth medium (EGM^®^-2; Clonetics; Lonza Cologne GmbH, Cologne, Germany) supplemented with the EGM^®^-2 BulletKit^®^ [hydrocortisone, 0.4% human fibroblast growth factor-basic (hFGF-b); 0.1% vascular endothelial growth factor (VEGF); 0.1% recombinant analogue of insulin-like growth factor-1(R^3^-IGF-1); 0.1% ascorbic acid; 0.1% heparin; 2% fetal bovine serum (FBS); 0.1% hEGF and 0.1% gentamicin sulfate and amphotericin-B (GA-1000); incubation: 37 °C, 5% CO_2_] (Clonetics, Lonza Cologne GmbH, Cologne, Germany). The cells were used at passages 2–5, according to the manufacturer’s instructions.

### Dose-response evaluation

Endothelial cells were exposed to different concentrations of citrate (0.25, 0.8, 1.0, 1.5, 2.0 and 5.0 mM) and gluconate (0.25, 0.8, 1.0, 1.5, 2.0 and 5.0 mM) for 48 h. The proportions of living cells were evaluated by the neutral red (NR) uptake assay.^27^

### Sample preparation

Endothelial cells were cultured in six-well plates (BD Biosciences, Stockholm, Sweden) until approximately 80% confluent. The cells were treated with 30 mM D-glucose (Merck KGaA, Darmstadt, Germany) to mimic the state of hyperglycaemia or 50 µM 3,4-DGE, which was extracted from glucose-containing fluid according to Linden et al.,^28^ alone or with the addition of 0.8 mM citrate (trisodium citrate dihydrate; Merck KGaA, Darmstadt, Germany) or a combination (henceforth referred to as the ‘CAG combination’) of 0.8 mM citrate and 1 mM gluconate (sodium gluconate; Jungbunzlauer AG, Basel, Switzerland) dissolved in supplemented EGM-2 medium followed by incubation (37 °C, 5% CO_2_) for 48 h. GDP concentration was chosen according to previous studies^8^ and the concentrations of citrate and the CAG combination were selected after dose-response studies (supplementary Figure 1). Endothelial cells treated with EGM-2 medium were used as a negative control. As an additional control, the cells were also treated with 0.8 mM citrate or with the CAG combination.

### Detection of apoptosis and necrosis

After incubation with different combinations, the cells were detached [trypsin-ethylenediamine tetraacetic acid (EDTA) for about 5 min at room temperature, followed by the addition of a trypsin inhibitor], washed in phosphate-buffered saline (PBS; twice, 5 min, 1000 rpm) and stained with annexin V-Alexa Fluor^®^488 (Life Technologies Europe BV, Stockholm, Sweden) (1:100, 15 min in the dark on ice) to detect apoptosis and 7-aminoactinomycin D (7-AAD; BD Via-Probe, BD Pharmingen Biosciences, San Diego, California, USA) (1:100, 15 min in the dark on ice) to test for late apoptosis and necrosis. The fluorescence was evaluated using a EPICS^®^ XL-MCL^™^ flow cytometer (Beckman Coulter Inc., Brea, California, USA), and fluorescence intensity was standardised using Flow-Set fluorospheres (Beckman Coulter Inc., Brea, California, USA).

Apoptosis was further visualised by confocal microscopy. After incubation with different combinations, the cells were harvested on glass slides in a cytospin2 centrifuge (RP centrifuge; Hettich Rotanta, Malmö, Sweden) at 600 rpm for 5 min and fixed in 100% methanol. Before staining, the cells were permeabilised with 0.25% Triton-X 100 (VWR International, Göteborg, Sweden) in PBS with 5% FBS for 10 min. Slides were incubated with anti-chemokine (C-X-C motif) receptor 2 [CXCR2; interleukin-8 receptor, beta (IL8RB) antibody or control immunoglobulin G (IgG) antibody (10 µg/ml; mouse IgG isotype control; R&D Systems, Copenhagen, Denmark)] for 40 min at room temperature. The slides were washed in PBS and incubated with secondary goat anti-mouse IgG antibodies (Alexa Fluor^®^ 488, Invitrogen Corporation, Carlsbad, California, USA) in a dilution of 1:200 for 40 min in the dark at room temperature. Staining of DNA was performed by incubating the cells with propidium iodide (1 mg/ml) for 15 min in the dark at room temperature. After washing in PBS, the slides were examined with the LSM 510 confocal laser scanning microscope (Carl Zeiss MicroImaging GmbH, Göttingen, Germany).

### PKC activation

Cells were treated with glucose/3,4-DGE and citrate/CAG as previously described and washed with PBS containing 0.2 mM phenylmethylsulfonyl fluoride (PMSF), 1 µg/ml pepstatin A, 5 µg/ml leupeptin (Sigma-Aldrich Sweden AB, Stockholm, Sweden) and a complete protease inhibitor cocktail (Roche Diagnostics GmbH, Mannheim, Germany) and lysed in modified radio immunoprecipitation assay (RIPA) [50 mM 2-[4-(2-hydroxyethyl)piperazin-1-yl]ethanesulfonic acid (HEPES), 150 mM NaCl, 2 mM EDTA, 50 mM ZnCl_2_, 1% nonyl phenoxylpolyethoxylethanol (NP-40), 0.1% sodium deoxycholate, 0.1% sodium dodecyl sulfate (SDS) containing the same protease inhibitors]. Protein concentrations were measured with the detergent compatible (DC™) protein assay (Bio-Rad Laboratories, Hercules, California, USA). Equal amounts of protein were separated by SDS-polyacrylamide gel electrophoresis (PAGE) and blotted onto polyvinylidene fluoride (PVDF) membranes. Membranes were saturated with non-fat dry milk or bovine serum albumin (BSA) and incubated with mouse monoclonal antibodies against PKC-βII (Millipore AB, Solna, Sweden) (all 1:500–1000) or mouse anti-glyceraldehyde 3-phosphate dehydrogenase (GAPDH) antibody as loading control (1:3000–5000; Novus Europe, Cambridge, UK). Bound antibodies were detected with rabbit anti-mouse horseradish peroxidase (HRP)-conjugated antibody (1:50,000–200,000; Novus Europe, Cambridge, UK) using the ECL Plus Western blotting reagent (GE Healthcare UK Ltd, Little Chalfont, UK) and the GelDoc equipment (Bio-Rad Laboratories AB, Sundbyberg, Sweden). To quantify protein levels, band intensity was measured with the ImageJ software version 1.24 (National Institutes of Health, Bethesda, Maryland, USA) and normalised against GAPDH. If required, membranes were stripped with Restore Western Blot Stripping Buffer (Thermo Fisher Scientific Inc., Rockford, Illinois, USA), blocked and reprobed with new antibodies.

### ICAM-1 expression

After exposure to different combinations, the endothelial cells were enzymatically detached according to the manufacturer’s instructions and washed in PBS (twice, 5 min, 1000 rpm) followed by the addition of antibodies for expression of the VCAM-1 (10 µg/ml) or ICAM-1 (10 µg/ml) adhesion molecules (R&D Systems, Copenhagen, Denmark). Cells were incubated in the dark on ice for 30 min, then washed in PBS (twice, 5 min, 1000 rpm), and stained with secondary rabbit anti-mouse IgG-fluorescein isothiocyanate (FITC) rabbit f(ab′)_2_ antibody (10 µg/ml) (Dako Sweden AB, Stockholm, Sweden) and incubated for 15 min in the dark on ice. After an additional washing step (PBS, twice, 5 min, 1000 rpm) the expression of ICAM-1 and VCAM-1 was analysed using the EPICS^®^ XL-MCL™ flow cytometer (Beckman Coulter Inc., Brea, California, USA), and fluorescence intensity was standardised using Flow-Set fluorospheres (Beckman Coulter Inc., Brea, California, USA). An IgG negative control (Life Technologies Europe BV, Stockholm, Sweden) was used as a negative control.

### Neutrophil migration

HUVECs were grown on Transwell^®^ inserts (12-well, 3 µM pore size; Sigma-Aldrich Chemie GmbH, Munich, Germany) in EGM-2 medium, as previously described.^29^ As a control, cell viability was analysed by trypan blue exclusion assay according to the manufacturer’s instructions (Sigma-Aldrich Chemie GmbH, Munich, Germany). The cell growth medium was replaced with medium containing glucose, 3,4-DGE or combinations of glucose/3,4-DGE and citrate/CAG as in the experiments above, and the cells were incubated for 48 h. Cells treated with medium and cells treated with 0.8 mM citrate or the CAG combination were used as negative controls. Human neutrophil isolation and transmigration assays were performed as previously described.^29^ Briefly, 0.5 ml fresh culture medium was added to the top well, and fresh medium or medium containing the different combinations and *Escherichia coli* (10^8^ bacteria/cell, 1.5 ml) was added to the bottom well.^8,29^ The cluster plates were then incubated at 37 °C and at 5% CO_2_ atmosphere for 24 h, a time that induced maximal neutrophil migration across the endothelial monolayer. Neutrophils (1.5 × 10^6^, 0.5 ml) were added to the top well. After 3 h, samples were taken from the bottom well for neutrophil counting by Cell Counter (Sysmex UK Ltd, Milton Keynes, UK) and analysed for cytokines.

### Cytokine secretion

Secretion of IL-6 and CXCL8 by the infected endothelial cells was quantified in supernatants by enzyme-linked immunosorbent assay (ELISA; R&D Systems, Sweden) according to the manufacturer’s instructions.

### Statistical analysis

All data are shown as mean ± SEM. Each experiment was repeated 3–4 times. The statistical difference was investigated using the Mann–Whitney U test (***p ≤ 0.001, **p < 0.01, *p < 0.05, ns = not significant).

## Results

### Evaluation of optimal concentrations

To determine optimal antioxidant concentrations, we analysed the effect of the antioxidants on cell survival by dose-response curve studies (supplementary Figure 1). Endothelial cell viability was not affected by citrate or gluconate concentrations up to 5 mM as determined by the NR uptake assay.

### Citrate treatment decreases cell mortality

**Figure 1.**
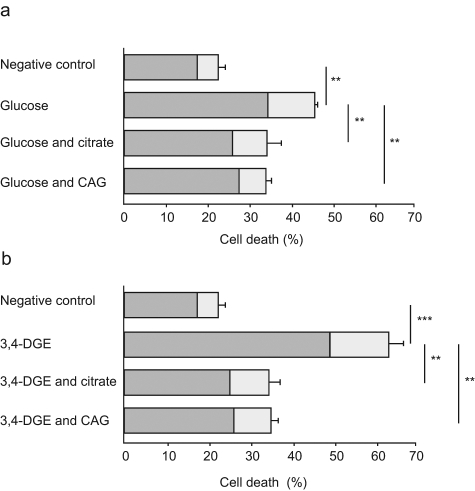
Citrate and citrate/gluconate decrease cell death. Glucose (a) and 3,4-dideoxyglucosone-3-ene (3,4-DGE) (b) treatment of endothelial cells increased total cell death. The figure shows the percentage of total cell death as well as the parts that are apoptotic (light grey) and necrotic (dark grey). Citrate and the 0.8 mM citrate and 1 mM gluconate (CAG) combination reduced apoptosis and necrosis after 48 h of incubation together with glucose (a) or 3,4-DGE (b). Glucose or 3,4-DGE treatment was compared to medium control, and citrate and CAG combination treatment of glucose and 3,4-DGE-damaged cells was compared to the respective additive. The results are the mean (± SEM) of four different experiments (a and b) (***p < 0.001, **p < 0.01).

To investigate whether citrate or the combination of citrate and gluconate influence the cause of cell death, endothelial cells were exposed to glucose and 3,4-DGE in the presence or absence of citrate or CAG combination (Figure 1). Natural cell death (medium control) was 22%, of which 5% was caused by apoptosis and 17% was caused by necrosis. Incubation of the endothelial cells with glucose or 3,4-DGE significantly increased the total number of dead cells to 46% and to 63% respectively compared to control (Figure 1a and 1b).

**Figure 2.**
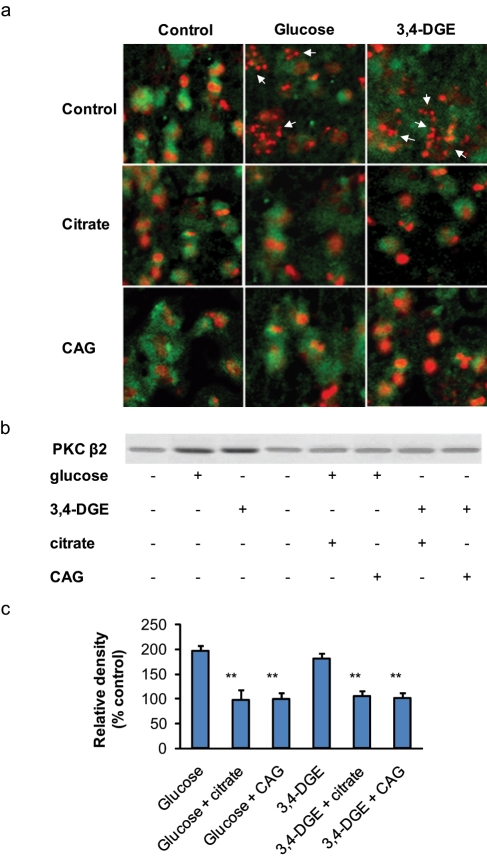
Citrate and citrate/gluconate decrease apoptosis and PKC-βII expression. (a) Confocal microscopy revealed a significant increase of nuclear fragmentation in glucose- or 3,4-DGE-treated epithelial cells compared to control (indicated by the arrows). The cellular membrane was stained green for chemokine (C-X-C motif) receptor 2 (CXCR2) with primary mouse antibodies followed by labelling with secondary antibodies conjugated with fluorescein isothiocyanate (FITC) (R&D Systems, Copenhagen, Denmark). Cell nuclei were stained with propidium iodide (red). Original magnification × 300. (b and c) Western blot analysis revealed a continuous basal PKC-βII expression in endothelial cells. Treatment with glucose or 3,4-DGE increased the amount of PKC-βII proteins. Citrate or 0.8 mM citrate and 1 mM gluconate (CAG) treatment of hyperglycaemia-damaged cells decreased PKC-βII expression down to background level. Quantification of band intensity was performed using the ImageJ version 1.24 software (National Institutes of Health, Bethesda, Maryland, USA) and normalised to glyceraldehyde 3-phosphate dehydrogenase (GAPDH) protein level. The results are the mean of three different experiments (± SEM) (**p < 0.01).

Glucose treatment of HUVECs increased the fraction of both apoptotic and necrotic cells (up to 11% and to 34%), while 3,4-DGE-treated HUVECs increased higher fractions of both apoptotic and necrotic cells (up to 14% and to 49%) compared to the control (Figure 1a and Figure 2a). The presence of citrate or CAG combination decreased the glucose-induced cell death drastically. Citrate treatment decreased the apoptotic fraction down to the level of the control (6%), as did the CAG combination which decreased glucose-induced cell death to 5%. Glucose-induced necrosis was decreased to 26% and 27% with citrate and CAG combination, respectively.

The treatment of 3,4-DGE-damaged endothelial cells with citrate or CAG combination significantly reduced total cell death to 35% (Figure 1b). The effect was most prominent on 3,4-DGE-induced necrosis, which was reduced to 25% with citrate treatment and to 26% with the CAG combination. 3,4-DGE-induced endothelial apoptosis was reduced to 9% with both citrate treatment alone or the CAG combination (Figure 1b and Figure 2b). The addition of citrate or CAG combination to control endothelial cells did not affect cell viability (data not shown).

### Citrate decreases PKC-βII expression

PKC activation is involved in the development of vascular dysfunction and plays a key role in the pathophysiology of diabetic vascular complications. Endothelial treatment with glucose or 3,4-DGE increased PKC-βII expression compared to unstimulated cells (Figure 2b and 2c). The addition of citrate or CAG combination during endothelial treatment with glucose or 3,4-DGE kept PKC-βII expression down to background level.

### Citrate decreases endothelial ICAM-1 expression

**Figure 3.**
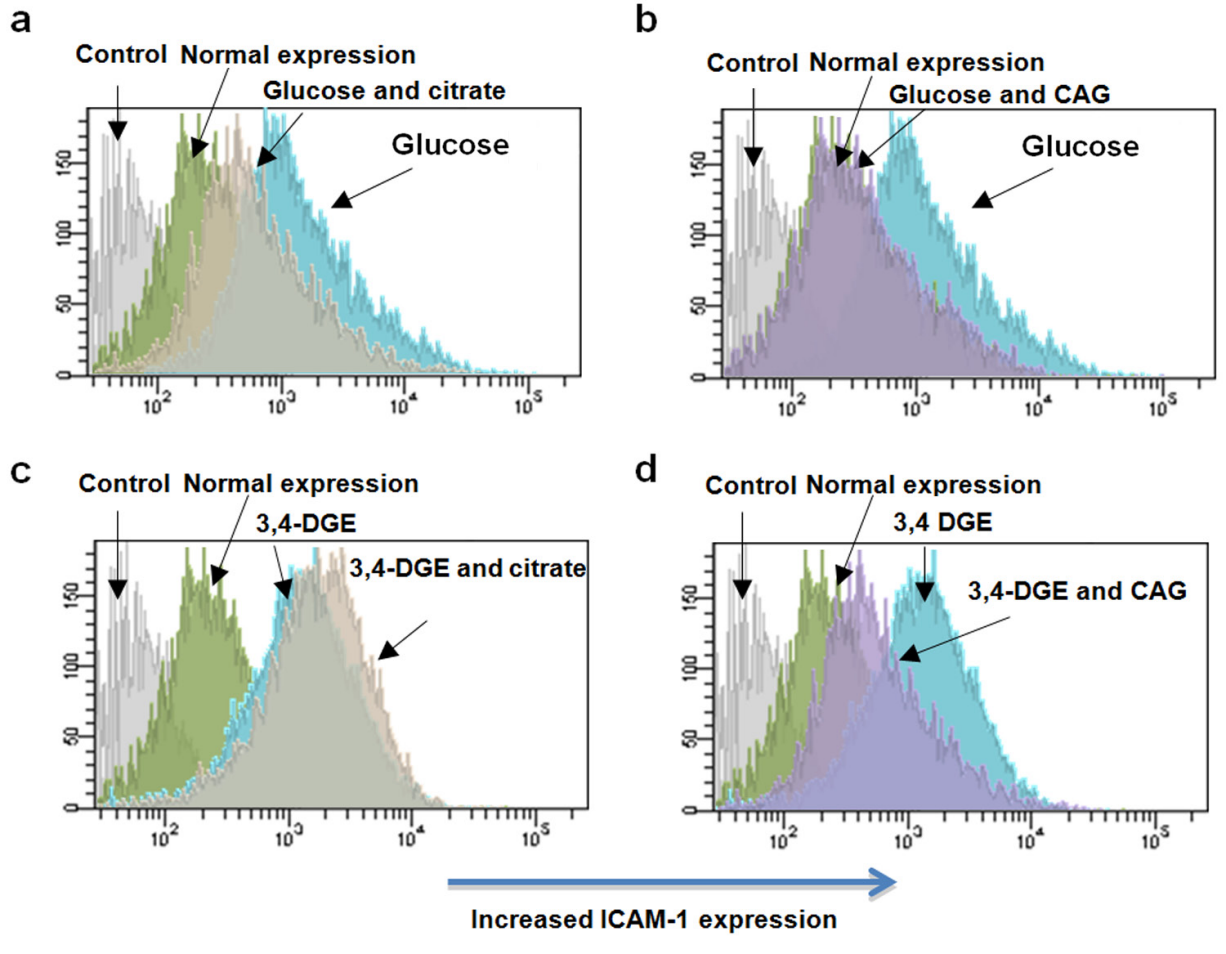
Citrate and the 0.8 mM citrate and 1 mM gluconate (CAG) combination decrease intercellular adhesion molecule-1 (ICAM-1) expression. ICAM-1 expression was analysed by flow cytometry on glucose or 3,4-DGE-treated endothelial cells and in the damaged cells in the presence of citrate or the CAG combination. Endothelial ICAM-1 expression after treatment with glucose and citrate (a), glucose and CAG combination (b), 3,4-DGE and citrate (c) and 3,4-DGE and CAG combination (d). Immunoglobulin G (IgG) negative control was used as the control. ICAM-1 expression was significantly upregulated by glucose or 3,4-DGE treatment (shift to the right). Addition of the CAG combination significantly decreased glucose or 3,4-DGE-induced ICAM-1 expression (shift to the left), while the addition of citrate only decreased glucose-induced ICAM-1 expression. The results are from one representative experiment out of a total of four experiments.

We investigated if citrate treatment of endothelial cells during conditions of hyperglycaemia affects ICAM-1 and VCAM-1. Exposure of endothelial cells to glucose or 3,4-DGE significantly upregulated ICAM-1 expression (Figure 3 and Table 1), but did not affect VCAM-1 expression (data not shown). Glucose-induced ICAM-1 expression was significantly reduced with the addition of citrate or CAG combination. ICAM-1 expression was also reduced by the presence of CAG combination in 3,4-DGE-damaged cells. However, gluconate alone did not reduce glucose or 3,4-DGE-increased endothelial ICAM-1 expression, and citrate alone did not reduce 3,4-DGE-increased endothelial ICAM-1 expression. Treatment of control endothelial cells with citrate, gluconate or CAG combination did not increase ICAM-1 expression (data not shown).

**Table 1.** ICAM-1 expression on endothelial cells

Additive to the cells	Mean^a^	± SEM	*p* value
None	8.8	0.08	–
Glucose	9.5	0.01	< 0.01^b^
Glucose and citrate	9.0	0.04	< 0.01^c^
Glucose and citrate/gluconate	8.7	0.01	< 0.01^c^
3,4-DGE	9.8	0.04	< 0.05^b^
3,4-DGE and citrate	9.6	0.01	ns^d^
3,4-DGE and citrate/gluconate	8.5	0.04	<0.05^e^

3,4-DGE: 3,4-dideoxyglucosone-3-ene, ICAM-1: intercellular adhesion molecule-1.

a The results are the mean (± SEM) of four separate experiments; ^b^compared to the negative control; ^c^compared to glucose-treated cells; ^d^compared to 3,4-DGE- and citrate-treated cells (ns = not significant); ^e^compared to 3,4-DGE- and citrate/gluconate-treated cells.

### Citrate decreases neutrophil migration across infected cells

**Figure 4.**
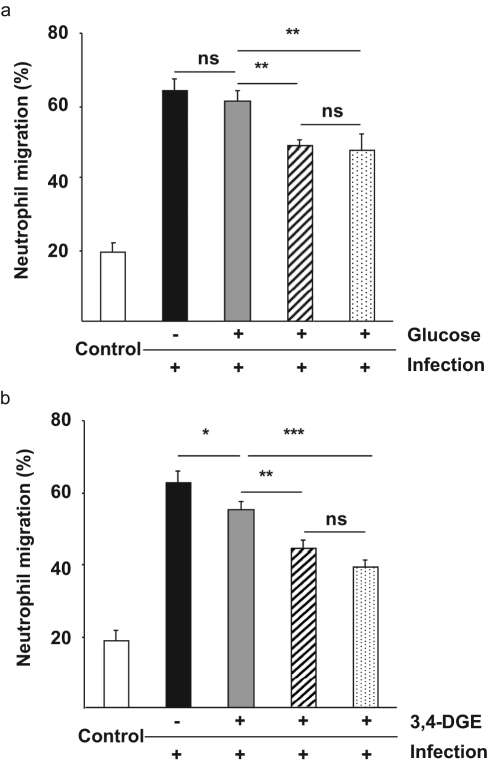
Citrate and the 0.8 mM citrate and 1 mM gluconate (CAG) combination decrease neutrophil diapedesis across infected cells. Endothelial cells were grown in glucose (grey) (panel a) or 3,4-DGE (grey) (panel b) alone or in the presence of citrate (dashed) and CAG combination (dotted) for 2 days before the addition of human neutrophils to the upper compartment of activated monolayers. Migrated neutrophils were calculated in the lower compartment after 3 h. (a) The addition of citrate and CAG combination decreased neutrophil migration across infected endothelial cells, but glucose treatment did not have any influence. (b) The 3,4-DGE treatment of endothelial cells had a small impact on neutrophil migration that was further reduced by citrate or CAG combination treatment. Cells grown in medium alone were used as a negative control (white) and untreated infected cells were used as positive control (black). The results are the mean (± SEM) of three different experiments (***p < 0.001, **p < 0.01, *p < 0.05, ns = not significant).

To investigate how glucose and 3,4-DGE affect endothelial cell function, we cultured HUVECs in the Transwell^®^ model and investigated if infection-induced neutrophil diapedesis could be affected by glucose (Figure 4a) or 3,4-DGE (Figure 4b) in the presence or absence of citrate or CAG combination. Infection increased neutrophil diapedesis by 48%, but was not further affected by glucose or 3,4-DGE treatment. However, neutrophil migration was significantly reduced by citrate or CAG treatment of infected and glucose or 3,4-DGE-treated endothelial cells (*p* < 0.01 and *p* < 0.001). Treatment of infected endothelium with citrate or CAG combination reduced neutrophil diapedesis to the same extent, while neither citrate nor CAG combination treatment of non-infected control cells had any effect (data not shown).

### Citrate decreases inflammation

**Figure 5.**
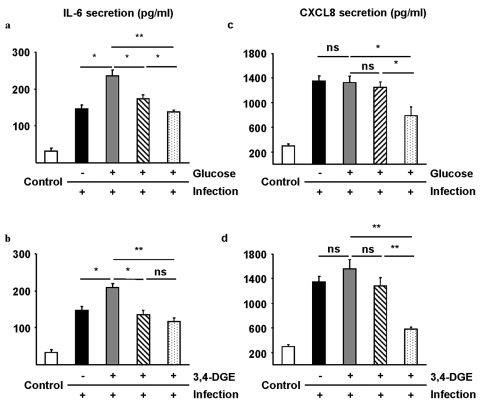
Citrate and 0.8 mM citrate and 1 mM gluconate (CAG) combination decrease inflammation. Secretion of interleukin-6 (IL-6) (a and b) and chemokine (C-X-C motif) ligand 8 (CXCL8) (c and d) from endothelial cells treated with glucose (grey) (panel c) or 3,4-DGE (grey) (panel d) alone or in the presence of citrate (dashed) or CAG combination (dotted). The presence of citrate and CAG combination decreased glucose- and 3,4-DGE-induced IL-6 secretion (a and b). CXCL8 secretion was not affected by glucose or 3,4-DGE treatment of endothelial cells, but the presence of citrate decreased endothelial CXCL8 secretion (c and d). Cells grown in medium alone were used as a negative control (white) and untreated infected cells were used as positive control (black). The results are the mean (±SEM) of three different experiments (**p < 0.01, *p < 0.05, ns = not significant).

Endothelial cells were treated with glucose or 3,4-DGE with or without citrate or CAG combination to further investigate the effect of glucose and 3,4-DGE on endothelial dysfunction during inflammation. Secretion of the proinflammatory cytokine, IL-6, and the chemotactic cytokine, CXCL8, was used as a model of inflammation. Basal IL-6 secretion (negative control) was 33 pg/ml (Figure 5a and 5b). An infection significantly increased IL-6 secretion to 146 pg/ml and IL-6 secretion increased even further to 235 pg/ml with glucose (Figure 5a) and to 208 pg/ml with 3,4-DGE (Figure 5b). Using citrate alone on infected endothelial cells in the presence of glucose or 3,4-DGE reduced IL-6 secretion to 173 and 135 pg/ml and the CAG combination reduced cytokine secretion even further to 138 and 118 pg/ml, respectively (Figure 5a and 5b). The CAG combination significantly decreased the level of IL-6 secretion from non-infected endothelial cells while citrate treatment alone had a lesser effect, 138 pg/ml and 173 pg/ml, respectively (*p* = 0.048, data not shown).

Endothelial cells secrete the neutrophil chemokine CXCL8 upon infection. The basal level of CXCL8 in control samples was 295 pg/ml, which increased to 1354 pg/ml upon infection. Glucose or 3,4-DGE treatment of infected endothelial cells increased CXCL8 secretion slightly more, but the increase was not significant. Citrate treatment of infected cells exposed to glucose or 3,4-DGE did not decrease CXCL8 secretion significantly. However, the CAG combination significantly decreased CXCL8 secretion from infected cells exposed to glucose or 3,4-DGE to 788 and 576 pg/ml, respectively (*p* < 0.05 and *p* < 0.01 respectively).

## Discussion

Endothelial dysfunction is an early manifestation of vascular atherosclerosis and cardiovascular disease. Confirming previous studies, we found that hyperglycaemia and 3,4-DGE significantly increased both necrosis and apoptosis, with 3,4-DGE causing the highest cell death. To evaluate the capacity of citrate and gluconate on endothelial survival, hyperglycaemia-damaged endothelial cells were treated with these compounds and analysed for apoptosis and necrosis. Remarkably, we found that endothelial treatment with citrate or with the CAG combination decreased hyperglycaemia and 3,4-DGE induced cell death by up to 50%. The mechanism behind citrate-induced reduction in cell death was shown to involve a reduced PKC-βII expression. PKC-βII is a member of the superfamily of serine/threonine kinases that respond to Ca^2+^ and diacylglycerol (DAG) for downstream signalling.^30^ To be activated, PKC must be properly phosphorylated, but while phosphorylations at the activation site are dispensable for activity, phosphorylation of the turn motif is absolutely required to maintain catalytic competence of the enzyme.^31^ Supporting our observations, blocking of the PKC pathway was previously shown to decrease hyperglycaemia-caused apoptosis.^32^ Citrate is also a well-known Ca^2+^ chelator and could thus decrease PKC activation by blocking the Ca^2+^-mediated translocation of PKC from the cytosol to the membrane. Several clinical studies on vascular function in diabetic patients have shown that inhibition of the PKC-β pathway has beneficial effects on vascular blood flow.^33,34^ However, the blocking of PKC-β did not affect the increased oxidative state in diabetic patients,^34,35^ suggesting that other isoforms of PKC, including alpha and delta, may participate in vascular dysfunction.^36^

Hyperglycaemia-induced expression of adhesion molecules and the modulation of junctional permeability is one of the initial events of endothelial dysfunction and was recently shown to be mediated by the PKC pathways.^19,37^ In this study, both hyperglycaemia and 3,4-DGE were found to increase endothelial ICAM-1 expression, but the expression of VCAM-1 was not affected. Citrate treatment decreased both hyperglycaemia-induced ICAM-1 expression and hyperglycaemia-induced neutrophil diapedesis. However, citrate treatment did not decrease 3,4-DGE-induced ICAM-1 expression, although 3,4-DGE-induced neutrophil diapedesis was decreased by citrate treatment. This discrepancy between ICAM-1 expression and neutrophil diapedesis could indicate that citrate affects additional mechanisms involved in neutrophil diapedesis. On the other hand, the CAG combination exhibited a highly significant decrease of both hyperglycaemia- and 3,4-DGE-induced ICAM-1 expression. Furthermore, the combination significantly decreased both hyperglycaemia and 3,4-DGE-induced neutrophil diapedesis. These findings supports a previous report indicating that citrate improves the effect of other antioxidants.^38^

A recent study in septic patients with hyperglycaemia showed increased neutrophil chemotaxis due to an increase of adhesion molecules.^39^ Furthermore, chemokine-driven leukocyte invasion of hyperglycaemic dysfunctional endothelium gives rise to inflammation and plaque formation.^40^ We observed only a minor increase of IL-6 and CXCL8 from hyperglycaemia- or 3,4-DGE-damaged cells in the infection model. Treatment with citrate significantly decreased IL-6 secretion from infected cells, but CXCL8 secretion was not significantly affected. However, the CAG combination decreased both cytokines, although the combination had the most impact on 3,4-DGE-induced CXCL8 secretion. Apart from the chelating capacities of citrates and gluconates, the positive effects of these compounds can originate from their quality as free radical scavengers,^41,42^ thereby blocking hyperglycaemia-induced ROS production.^7,32,43^ Additionally, citrate is known to maintain the glutathione (GSH)/oxidised glutathione (GSSG) ratio, the central component of the myocardial antioxidant system (reviewed by Mallet et al.).^38^

To the best of our knowledge, this is the first study analysing the impact of citrate treatment on hyperglycaemia-damaged endothelial cells. Here we demonstrate that citrate treatment alone, or in combination with gluconate, decreases PKC-IIβ expression and mitigates both necrosis and apoptosis in hyperglycaemia- or 3,4-DGE-damaged cells. Citrate treatment was also shown to decrease endothelial inflammation by reducing ICAM-1 expression and cytokine production. Furthermore, the presence of citrate during hyperglycaemic conditions was shown to improve endothelial function by decreasing neutrophil diapedesis. Although very promising, these *in vitro* results should be correlated with *in vivo* studies using animal models. In summary, these results suggest that citrate alone or in combination with gluconate can aid potential therapeutic approaches to endothelial dysfunction induced by hyperglycaemia.
